# A Digital Signal Processing-Based Multi-Channel Acoustic Emission Acquisition System with a Simplified Analog Front-End

**DOI:** 10.3390/s25103206

**Published:** 2025-05-20

**Authors:** Gan Tang

**Affiliations:** School of Mechatronics and Vehicle Engineering, East China Jiaotong University, Nanchang 330013, China; gan_tang@ecjtu.edu.cn

**Keywords:** acoustic emission, multi-channel data acquisition, digital signal processing, analog front-end, LabVIEW, real-time signal processing, structural health monitoring

## Abstract

**Highlights:**

A simplified analog front-end is designed for multi-channel acoustic emission (AE) signal acquisition.

The system architecture reduces hardware complexity while maintaining measurement accuracy.

A DSP-based real-time processing algorithm enables efficient signal detection and compression.

Experimental results validate the performance across multiple AE channels under real-world conditions.

**What are the main findings?**

- A simplified analog front-end circuit can effectively support multi-channel AE signal acquisition without compromising signal integrity.
- The proposed DSP-based system enables the real-time processing and accurate characterization of AE signals across all acquisition channels.

**What are the implications of the main findings?**

- The simplified analog front-end design lowers system cost and complexity, making multi-channel AE monitoring more accessible for industrial applications.
- The real-time DSP-based processing approach provides a scalable foundation for intelligent structural health monitoring systems.

**Abstract:**

Advanced multi-channel acoustic emission (AE) monitoring systems often rely on complex and costly architectures, especially those requiring custom FPGA-based hardware. In this work, we present a digital signal processing (DSP)-based approach to high-performance AE acquisition, implemented using a simplified analog front-end (AFE) and a commercially available synchronous data acquisition (DAQ) card (NI USB-6356). This design eliminates the need for specialized FPGA development, improving accessibility and reducing system complexity. A key feature of the system is the replacement of traditional analog filters with a software-defined digital band-pass filtering module implemented in LabVIEW. This allows for real-time or post-processing filtering with adjustable parameters, enhancing flexibility in data analysis. The system supports 8-channel synchronous sampling at 1.25 MS/s, and performance evaluations demonstrate a dynamic range of 79.22 dB and a signal-to-noise ratio (SNR) of 85.39 dB. These results confirm the system’s ability to maintain high fidelity in AE signal acquisition without the need for dedicated hardware filtering or custom DAQ hardware. The proposed method offers a practical and validated alternative for multi-channel AE monitoring, with potential applications in structural health monitoring, materials testing, and other engineering domains.

## 1. Introduction

Acoustic emission (AE) sensing provides invaluable insights into the dynamic processes within materials and structures, underpinning critical applications in non-destructive testing (NDT), structural health monitoring (SHM), materials characterization, and geophysics [1]. As research frontiers expand toward understanding complex phenomena in large-scale or intricate systems, the demand for high-fidelity, multi-channel AE data acquisition has intensified. Such systems are essential for enhanced source localization [2,3,4], comprehensive damage assessment, and capturing spatially distributed transient events, with early explorations demonstrating the potential of multi-channel AE even in diverse fields like geotechnical engineering. However, the practical implementation of robust multi-channel AE systems faces significant instrumentation challenges that can impede broader scientific adoption and limit their deployment in demanding real-world scenarios.

Conventional approaches often rely on complex analog front-ends (AFEs) incorporating dedicated hardware filters for signal conditioning. While functional, this strategy typically results in systems that are inflexible, difficult to reconfigure for different experimental needs (making adaptation to specific material responses or changing noise conditions difficult), and potentially costly to scale, especially for applications requiring a large number of channels [5,6]. Alternatively, achieving higher performance and synchronization often necessitates sophisticated systems built around Field-Programmable Gate Arrays (FPGAs) [2,3]. Although powerful and capable of accelerating complex algorithms, FPGA-based solutions introduce substantial hurdles. Implementing designs often requires significant specialized hardware expertise using Hardware Description Languages (HDLs), a factor underscored by methodologies aiming to enable non-FPGA specialists to utilize these platforms via High-Level Synthesis (HLS) [7]. Even with HLS, successfully porting a complex code involves considerable refactoring effort and hardware-aware optimization [7]. Furthermore, the FPGA development cycle itself can be complex and time-consuming [7]. These factors regarding development complexity, required expertise, and potentially long development cycles contribute to higher overall costs and limit the accessibility of purely FPGA-based custom solutions. While commercial data acquisition platforms like those from National Instruments (NI) offer potential hardware foundations [6], effectively leveraging them for demanding multi-channel AE applications without succumbing to the pitfalls of designing either complex custom analog filtering hardware or intricate FPGA logic remains a challenge. A central difficulty lies in balancing the need for a simplified, potentially lower-cost AFE against the stringent low-noise and high-fidelity requirements inherent to AE measurements. This balance is crucial for enabling wider adoption in fields such as the condition monitoring of critical industrial machinery, like the fault diagnosis of tilting pad thrust bearings, where AE offers high sensitivity [8], or large-scale structural health monitoring, where optimizing the cost-effectiveness of deploying potentially numerous sensor channels is a major consideration, as highlighted by studies on sensor network optimization for structures like tall buildings [9]. Furthermore, as noted in several reviews [10,11,12,13,14,15], many existing system descriptions based on COTS platforms often lack comprehensive and transparent performance validation data. Consequently, a critical need exists for a more accessible, flexible, rigorously validated, yet high-performance methodology for multi-channel AE data acquisition.

To address this gap, we introduce and validate a novel approach centered on two key principles: (1) leveraging the power of digital signal processing (DSP) to implement core filtering functionalities in software, thereby enabling a significantly simplified AFE, and (2) building the system around a high-performance, commercially available synchronous DAQ card (NI USB-6356), thus deliberately bypassing the need for complex and costly bespoke FPGA development. Our methodology employs a standard low-noise preamplifier coupled directly to the commercial DAQ card, with versatile digital band-pass filtering implemented using custom LabVIEW software. This software-defined filtering offers unparalleled flexibility, allowing dynamic parameter adjustment and selection between real-time or post-processing modes, adapting readily to diverse AE signals and analytical workflows.

This paper details the design, implementation, and rigorous experimental characterization of this DSP-centric, multi-channel AE acquisition system. We present comprehensive performance evaluations covering sampling accuracy, noise characteristics, inter-channel consistency, linearity, dynamic range, and critically, overall synchronization performance under operational conditions. Furthermore, we demonstrate the system’s efficacy in capturing real-world AE signals via pencil lead break tests. By validating this streamlined yet powerful approach, we aim to provide the scientific community with a well-characterized, readily implementable technological resource. This methodology offers a pathway to high-fidelity multi-channel AE data acquisition that enhances flexibility and potentially lowers barriers to entry, facilitating advancements across the diverse scientific and engineering domains reliant on AE sensing, including practical applications in SHM, machinery diagnostics, NDT, and materials research [4,8,9,15].

## 2. System Design

**Figure 1:**
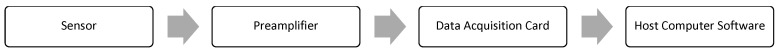
Schematic overview of the DSP-centric AE acquisition system architecture. Sensor: RS-2A (Ruandao Times Technology Co., Ltd., Beijing, China). Preamplifier: Custom-built (East China Jiaotong University, Nanchang, China). Data Acquisition Card: NI USB-6356 (National Instruments, Austin, TX, USA). Host Computer Software: LabVIEW 2015 (National Instruments, Austin, TX, USA).

We designed a multi-channel AE data acquisition system predicated on simplifying the analog hardware by leveraging software-based digital signal processing and reliable commercial off-the-shelf (COTS) components. This approach aims to enhance flexibility and accessibility compared to traditional hardware-centric or complex custom-FPGA systems. The overall system architecture follows a streamlined signal path: AE sensors capture the signals, which are amplified by a basic, low-noise preamplifier (Custom-built (East China Jiaotong University, Nanchang, Jiangxi, China)), before being digitized and synchronized by a high-performance commercial DAQ card. All subsequent filtering and processing occur in software on the host computer (Figure 1).

### 2.1. Hardware Implementation

The hardware configuration was deliberately kept minimal, focusing on high-fidelity signal capture and digitization, leaving complex filtering tasks to the software domain.

#### 2.1.1. AE Sensors

Standard RS-2A piezoelectric AE sensors were selected for their proven performance, offering excellent sensitivity within a broad frequency range (50 kHz–400 kHz) suitable for capturing typical AE transients generated during material deformation or fracture. Their compact size and wide operating temperature range facilitate deployment in various experimental setups.

#### 2.1.2. Preamplifier Stage

**Figure 2:**
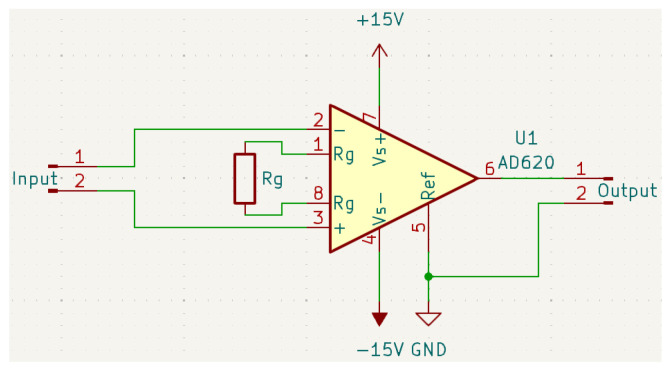
Preamplifier circuit schematic based on the AD620 instrumentation amplifier [16].

Consistent with our simplified AFE philosophy, the preamplifier stage focuses solely on low-noise amplification. A custom preamplifier was designed based on the Analog Devices AD620 instrumentation amplifier chip (Figure 2). This choice was driven by the AD620’s low input voltage noise (9 nV/√Hz), high common-mode rejection ratio (>80–100 dB), sufficient bandwidth (e.g., 120 kHz at G = 100, up to 1 MHz at G = 1), and ease of gain adjustment via a single external resistor (R_G_) [16]. This stage provides the necessary gain to match the weak AE signals to the DAQ input range but incorporates no hardware filtering, deferring frequency selection entirely to the digital domain. The adjustable gain allows optimization for different signal levels while managing noise contribution, a crucial consideration detailed in the performance evaluation (Section 3.1.2).

(1) $$R_{G}=\frac{49.4\;k\Omega }{G-1}$$

#### 2.1.3. Core Data Acquisition and Synchronization

A key element of our strategy is the use of a high-performance commercial DAQ card, specifically the National Instruments NI USB-6356 (an X Series device, National Instruments, Austin, TX, USA). This choice obviates the need for custom DAQ hardware development (e.g., FPGA-based systems), leveraging a well-characterized, reliable platform. The USB-6356 provides essential capabilities for multi-channel AE acquisition:

- High resolution and synchronous multi-channel capability: The 16-bit ADCs ensure accurate digitization. Critically, it supports simultaneous sampling across up to eight differential input channels, managed by the integrated NI-STC3 timing and synchronization technology. This built-in hardware synchronization ensures precise temporal correlation between channels, essential for AE analysis techniques like source localization [17].
- Sampling rate and anti-aliasing considerations: While the card supports a maximum sampling rate of up to 1.25 MS/s per channel, the primary AE validation experiments (Section 3.2) utilized 500 kS/s per channel. This rate was deliberately chosen as it sufficiently satisfies the Nyquist sampling theorem for the primary frequency range of the PLB AE signals observed (effectively capturing components up to 250 kHz), making the higher maximum rate unnecessary for these tests while aiding data management. It is important to note that the NI USB-6356 does not incorporate hardware anti-aliasing filters. Therefore, aliasing mitigation in this system relies on the combined effect of the RS-2A sensor’s limited nominal bandwidth (50 kHz–400 kHz), the bandwidth limitations of the AD620 preamplifier at the employed gain, and the fact that the dominant signal energy was well below the 250 kHz Nyquist frequency.
- Input impedance and interfacing: The NI USB-6356 features a very high input impedance (>100 GΩ||100 pF). This characteristic facilitates impedance bridging when connected to the low output impedance of the AD620 preamplifier (Section 2.1.2), ensuring minimal signal loading and accurate voltage transfer for digitization.
- Flexible input ranges: Software-selectable input ranges (±1 V to ±10 V) allow matching to the preamplifier output, optimizing the use of the ADC’s dynamic range.

**Figure 3:**
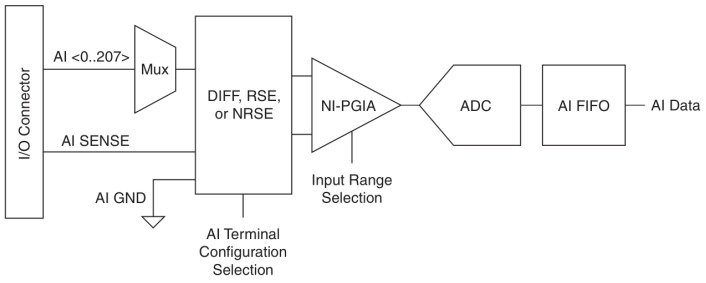
Simplified block diagram of the NI USB-6356 analog input path, showing key stages (PGA and ADC) [18].

Figure 3 provides a simplified functional block diagram of the card’s analog input path, illustrating key stages like the PGA and ADC.

#### 2.1.4. Data Interface

The NI USB-6356 utilizes a standard high-speed USB 2.0 interface (up to 480 Mbps) with an onboard buffer (32/64 Mb), providing sufficient bandwidth for continuous multi-channel data streaming to the host computer and simplifying system integration compared to other interface technologies like PCIe or PXI required by some high-end systems.

### 2.2. Software Design

The custom host software, developed in the LabVIEW graphical programming environment, is central to the system’s functionality, implementing crucial control, processing, and user interaction features. Its design prioritizes modularity, efficiency, and user control over acquisition and analysis parameters.

#### 2.2.1. Core Architecture

**Figure 4:**
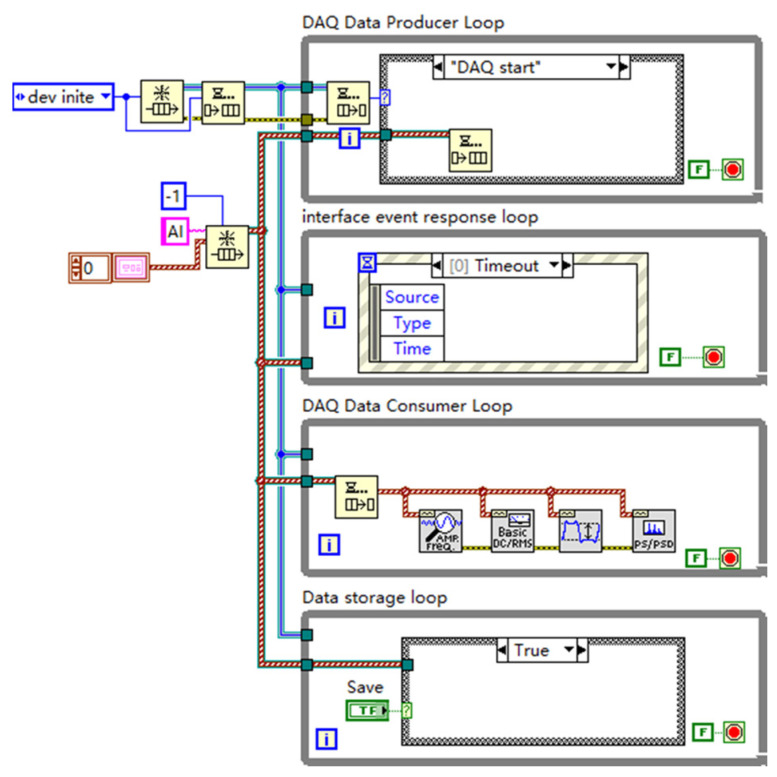
Schematic of the multi-loop producer–consumer software architecture enabling parallel data acquisition, processing, storage, and user interaction.

The software architecture is built upon the producer–consumer design pattern, extended into a multi-loop parallel structure (Figure 4) to handle high-speed, continuous data streams efficiently without data loss and maintain a responsive user interface. This architecture decouples the time-critical task of data acquisition from less time-sensitive tasks like processing, visualization, and storage.

**Figure 5:**
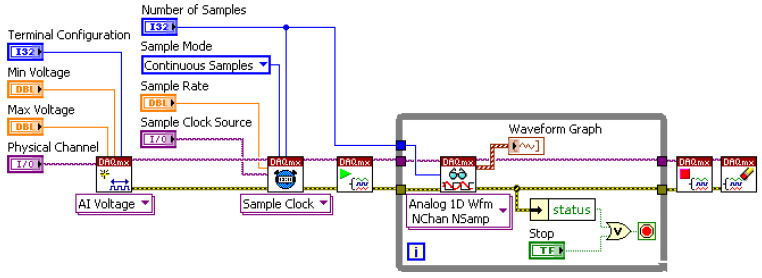
Example LabVIEW block diagram snippet showing DAQmx API usage for acquisition task configuration.

- DAQ Data Producer Loop: This loop interacts directly with the NI USB-6356 hardware via the NI DAQmx driver API (Figure 5) [19]. It handles device initialization and configuration (channels, sampling rate, range, hardware triggering) and continuously fetches acquired data blocks from the DAQ card’s buffer, placing them into a robust queue for consumption by other loops.
- DAQ Data Consumer Loop(s): One or more consumer loops retrieve data blocks from the queue. This is where core signal processing occurs, most importantly, the digital filtering (detailed below) but also potentially real-time feature extraction (e.g., amplitude, RMS), FFT-based spectral analysis (Power Spectrum, PSD), and preparation for visualization.
- Interface Event Response Loop: Handles all user interactions via the GUI (button presses and parameter changes), ensuring the system responds promptly to user commands without interrupting data flow.
- Data Storage Loop: Dedicated loop for writing raw or processed data to disk in selected formats (e.g., TDMS) for offline analysis and archival, ensuring data integrity even during high-throughput acquisition.

#### 2.2.2. Software-Defined Digital Filtering

**Figure 6:**
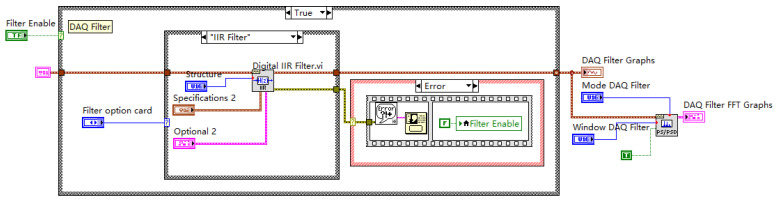
Illustration of the digital filtering implementation within the LabVIEW data processing module.

- A cornerstone of our simplified AFE approach is the implementation of band-pass filtering entirely within the LabVIEW software (typically executed in the DAQ Data Consumer Loop, Figure 6). This replaces inflexible hardware filters, offering significant advantages:
- Dynamic parameter adjustment: Filter characteristics (e.g., filter type, order, cutoff frequencies) can be easily modified through the GUI without any hardware changes, allowing rapid adaptation to different AE sources, materials, or noise environments. Users can select standard filter types like Butterworth, Chebyshev, etc. (e.g., 4th-order Butterworth used in validation tests).
- Real-time or post-processing: The filtering can be applied either in real-time to the incoming data stream (for immediate visualization or triggering) or applied/re-applied during the post-processing of the stored raw data, providing maximum analytical flexibility.
- Simplified hardware: Eliminates the need for complex analog filter circuits in the AFE.

#### 2.2.3. System Control and User Interface (GUI)

**Figure 7:**
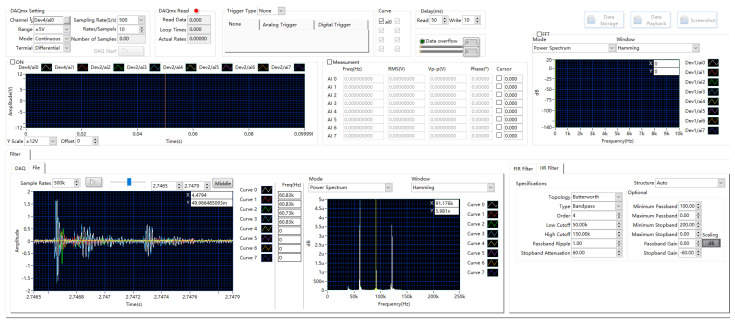
The main graphical user interface providing integrated control and visualization.

A user-friendly GUI (Figure 7) provides centralized control over all system operations and parameters. Developed in LabVIEW, it features distinct sections for the following:

- Function Control: Start/stop acquisition, save data, playback, screenshot, and status indicators.
- Acquisition Parameter Settings: Interactive configuration of channels, input ranges, sampling rate, acquisition duration/samples per channel, and trigger settings via the DAQmx API.
- Data Display: Real-time visualization of time-domain waveforms, frequency spectra (FFT), and key calculated parameters (e.g., RMS and peak amplitude), including interactive cursor measurements for quick analysis.

**Figure 8:**
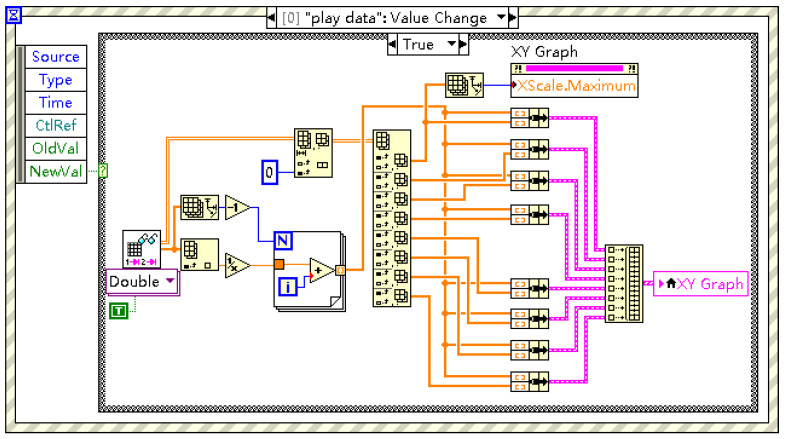
Example LabVIEW block diagram snippet illustrating event handling for system control.

The GUI and underlying system control logic (managed by the Interface Event Response and associated control loops, Figure 8) are designed for intuitive operation, simplifying experimental setup and monitoring.

## 3. System Performance Validation

A comprehensive suite of tests was conducted to rigorously validate the performance of the developed multi-channel AE acquisition system. The objectives were two-fold: first, to quantitatively characterize the system’s fundamental capabilities using standard electrical test signals, confirming the performance of the core COTS DAQ hardware (NI USB-6356) and the minimal AFE; second, to assess the system’s end-to-end functionality, including the software-defined digital filtering, in capturing representative AE signals. The evaluation encompassed key metrics critical for AE applications: sampling accuracy, noise characteristics, inter-channel consistency, linearity, dynamic range, and overall multi-channel synchronization under realistic operating conditions. While the inherent nanosecond-level hardware synchronization capabilities of the NI-STC3 technology within the DAQ card are well documented [17], this study focuses on evaluating the system-level synchronous performance achievable within the operational context (including USB interface and Windows host), acknowledging the practical limitations in directly measuring sub-microsecond timing errors in such environments without specialized external instrumentation. The results provide a robust characterization of the system as a deployable resource.

### 3.1. Basic Performance Indicator Testing 

#### 3.1.1. Sampling Rate and Frequency Accuracy

Accurate frequency capture over a wide band is fundamental for AE signal analysis. We assessed the system’s ability to faithfully acquire signals up to 500 kHz using various sampling rates provided by the NI USB-6356.

Method: Sine waves (1 Vpp) ranging from 1 kHz to 500 kHz were generated and directly input into the DAQ channels. Sampling rates (Fs) were set according to Table 1, generally using Fs ≥ 10f for f ≤ 125 kHz and the maximum Fs (1.25 MS/s) for higher frequencies to satisfy Nyquist criteria while characterizing performance across the card’s capabilities. Data (≥100 ms duration, averaged over 10 trials per setting) were acquired, and frequency content was analyzed using FFT.

**Figure 9:**
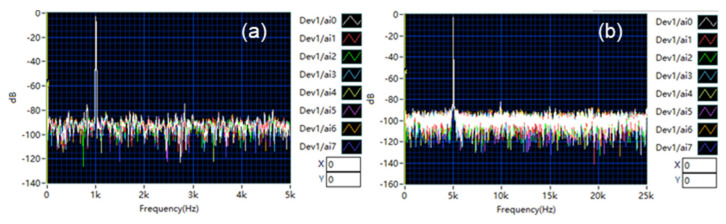

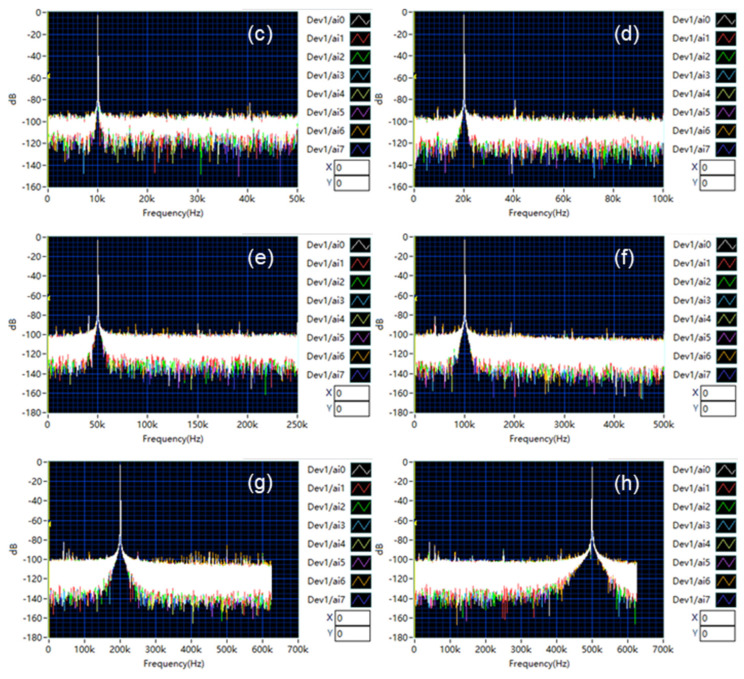
Representative FFT spectra demonstrating accurate signal captures at various frequencies. (**a**–**h**) correspond to the FFT analysis results when the experimental test signals were 1 kHz, 5 kHz, 10 kHz, 20 kHz, 50 kHz, 100 kHz, 200 kHz, and 500 kHz, respectively.

**Figure 10:**
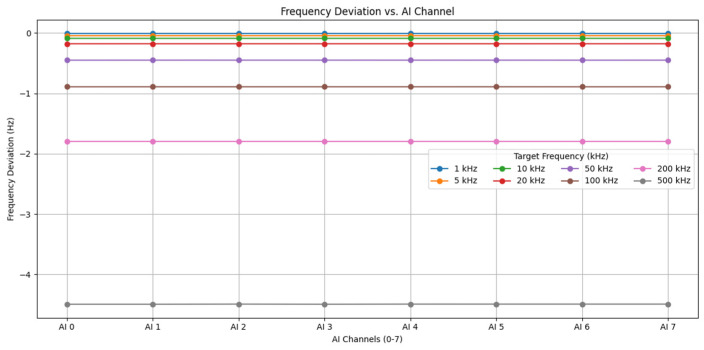
Frequency deviation analysis across channels and test frequencies.

Results and analysis: The system successfully captured all test frequencies, evidenced by clear peaks at the target frequencies in the spectra (Figure 9) with minimal spurious components. Frequency deviation analysis (Figure 10) demonstrated high accuracy, particularly at lower frequencies (deviation < 1 Hz at 1 kHz). While the absolute deviation increased modestly with frequency (reaching approx. −4.5 Hz at 500 kHz, Fs = 1.25 MS/s), the relative frequency error remained excellent, consistently below −0.01% across the tested range. Furthermore, inter-channel frequency measurements showed high consistency (e.g., <0.001 Hz difference between channels at 100 kHz). These results confirm that the core DAQ hardware provides accurate and consistent frequency digitization suitable for broadband AE signals, with the expected minor increase in deviation at higher frequencies likely reflecting inherent system limitations. The tests underscore the importance of selecting appropriate sampling rates (as per Table 1) for optimal performance.

#### 3.1.2. Noise Characterization

Understanding the system’s noise floor is crucial for determining its sensitivity to weak AE signals. We characterized the noise contributions from both the core DAQ hardware and the simplified preamplifier stage under various operating conditions.

Method: Noise levels were measured with inputs terminated to ground.

(1) DAQ baseline noise: To assess the inherent noise of the NI USB-6356, its differential inputs were directly shorted. Data were acquired across all channels, input ranges (±1 V to ±10 V), and sampling rates (1 kS/s to 1.25 MS/s, Table 2).

(2) System noise with preamplifier: To quantify the noise added by the AFE, the preamplifier input was grounded, its output connected to the DAQ inputs, and measurements repeated for preamplifier gains of 20 dB, 40 dB, and 60 dB (three trials per gain).

Noise magnitude was quantified using Root Mean Square (RMS) voltage over ≥1 s acquisition periods, and noise spectral distribution was examined via Power Spectral Density (PSD) plots derived from FFTs.

**Figure 11:**
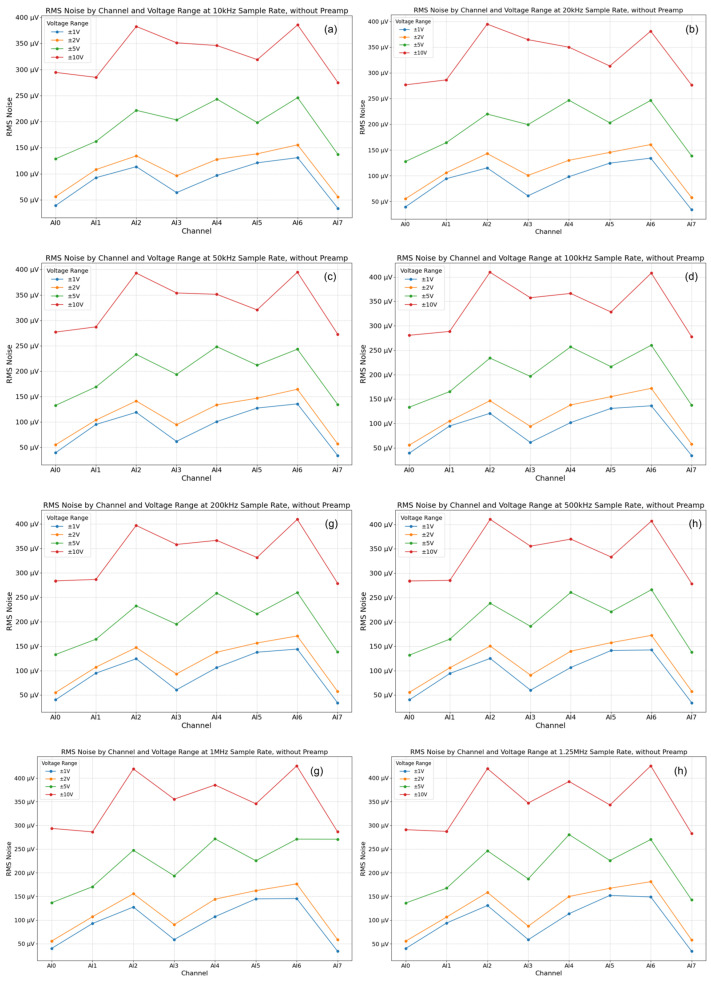
Baseline noise RMS voltage of the NI USB-6356 DAQ card (inputs shorted) across different channels, sampling rates, and input ranges. (**a**–**h**) correspond to the data of testing local noise at different sampling rates without using a preamplifier. The corresponding sampling rates are 10 kHz, 20 kHz, 50 kHz, 100 kHz, 200 kHz, 500 kHz, 1 MHz, and 1.25 MHz respectively.

**Figure 12:**
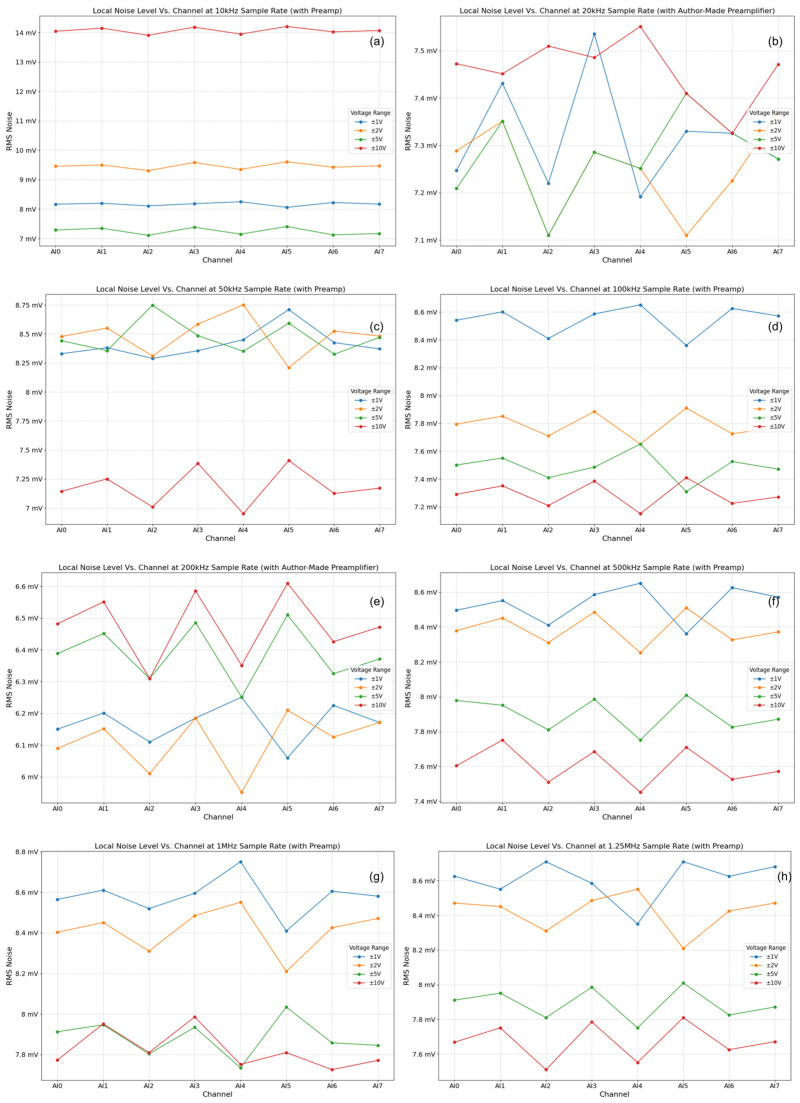
System noise RMS voltage with the preamplifier engaged (input grounded) at various gains. (**a**–**h**) show the system's background noise tested with the preamplifier connected, using different sampling rates. The sampling rates used for subfigures (**a**–**h**) were 1 kHz, 5 kHz, 10 kHz, 50 kHz, 100 kHz, 500 kHz, 1 MHz, and 1.25 MHz, respectively.

Results and analysis: The baseline noise of the NI USB-6356 card itself (inputs shorted, no preamplifier) was found to be low, generally in the µV RMS range, increasing slightly with higher sampling rates and larger input voltage ranges (Figure 11). This confirms the suitability of the COTS DAQ card for low-level signal acquisition. However, as anticipated, the preamplifier stage was identified as the dominant noise source in the complete system configuration (Figure 12). Engaging the preamplifier elevated the noise floor significantly, typically into the mV RMS range, particularly at higher gains (e.g., 60 dB). The noise level also exhibited dependence on the sampling rate, with higher rates integrating more noise bandwidth, an effect notably amplified when using the preamplifier (tens-to-hundreds-fold increase in noise power observed at 1.25 MHz with preamp vs. without). Minor variations in noise characteristics were observed between channels, likely due to inherent hardware tolerances. These results underscore a critical operational trade-off inherent to this simplified AFE design: while the preamplifier provides necessary gain for weak signals, its gain must be carefully selected in conjunction with the DAQ input range and sampling rate to achieve an optimal signal-to-noise ratio (SNR) for the specific AE signals being measured. The findings highlight the importance of minimizing preamplifier gain where possible and potentially implementing channel-specific noise assessment or compensation in software for the most demanding low-amplitude measurements.

#### 3.1.3. Inter-Channel Consistency: Frequency and Phase

Uniform performance across all acquisition channels is critical for multi-channel applications, like AE source localization, which rely on comparing signal arrival times or characteristics. We assessed the inter-channel consistency of the NI USB-6356 DAQ card in terms of both frequency and phase response.

Method: The test was conducted without the preamplifier to isolate the DAQ card’s intrinsic consistency. Sine wave signals (1 Vpp) spanning the frequency range (1 kHz–500 kHz, Table 3) were simultaneously applied to all eight input channels using appropriate signal splitting and impedance matching. The frequency and phase of the acquired signal on each channel were measured relative to the input signal or a reference channel.

**Figure 13:**
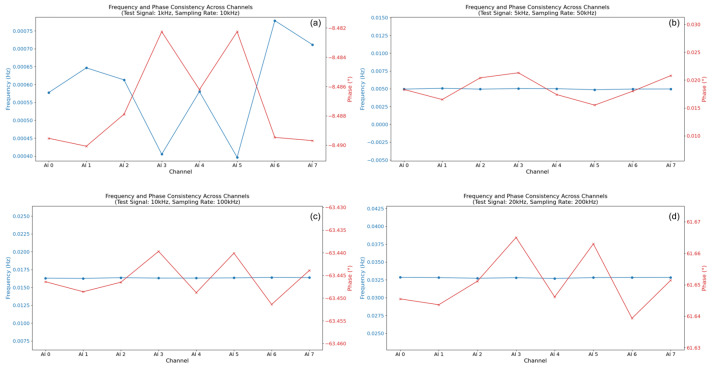

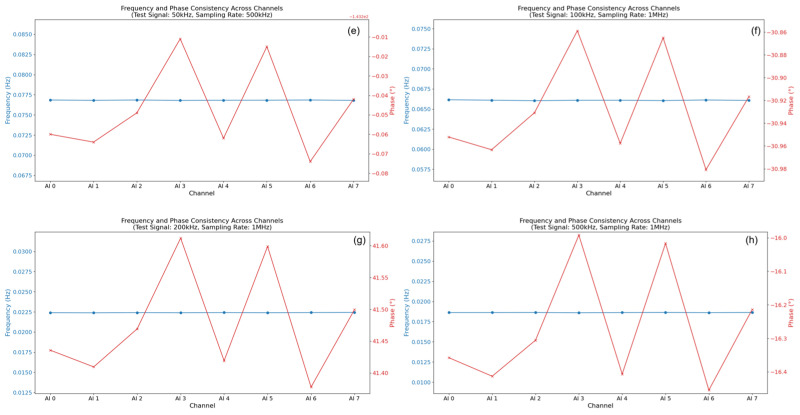
Inter-channel consistency results. (**a**–**h**) show the results of the system channel consistency tests performed with different test signal frequencies and corresponding sampling rates. The pairs are as follows: (**a**) 1 kHz test signal/10 kHz sampling rate. (**b**) 5 kHz test signal/50 kHz sampling rate. (**c**) 10 kHz test signal/100 kHz sampling rate. (**d**) 20 kHz test signal/200 kHz sampling rate. (**e**) 50 kHz test signal/500 kHz sampling rate. (**f**) 100 kHz test signal/1 MHz sampling rate. (**g**) 200 kHz test signal/1 MHz sampling rate. (**h**) 500 kHz test signal/1.25 MHz sampling rate.

Results and analysis: The system demonstrated excellent inter-channel consistency in frequency measurement (results shown in Figure 13). Across all tested frequencies, the measured frequency on each channel matched the input frequency with high fidelity (relative error < −0.01%), and the variation between channels was minimal (e.g., <0.001 Hz difference at 100 kHz). This confirms the synchronous and uniform operation of the ADCs and timing circuitry regarding frequency capture.

However, significant inter-channel variations were observed in the phase response, particularly as frequency increased. While the phase measurements tracked consistently within each channel across frequencies, the relative phase difference between channels became more pronounced at higher frequencies. For instance, at 500 kHz, phase differences corresponding to relative time delays on the order of nanoseconds to tens of nanoseconds were calculated (based on Δt ≈ Δφ/(2πf)). These phase/timing discrepancies likely originate from minute differences in signal path lengths, component delays, or clock skew within the DAQ card’s analog pathways and multiplexing circuitry preceding the ADCs. While inherent to many multi-channel systems, these results highlight that for applications demanding the highest timing precision (e.g., sub-microsecond accuracy for precise AE source localization), software-based phase/delay calibration and compensation are essential. The ability to implement such compensation readily in our LabVIEW software framework is a key advantage enabled by our DSP-centric approach, allowing correction for these inherent hardware non-uniformities.

#### 3.1.4. Amplitude Linearity

An accurate representation of signal amplitude across a wide range is essential for quantitative AE analysis. We assessed the linearity of the system’s amplitude response, focusing on the performance of the NI USB-6356 DAQ card itself.

Method: The test was conducted without the preamplifier. A 1 kHz sine wave was generated with precisely controlled peak-to-peak amplitudes (Vpp) ranging from 1 mV to 10 V (Table 4), covering the card’s available input ranges. The signal was applied to the input channels, and the acquired peak-to-peak amplitude was measured using the host software. Linearity was evaluated by comparing the measured Vpp to the known input Vpp across the tested range.

**Figure 14:**
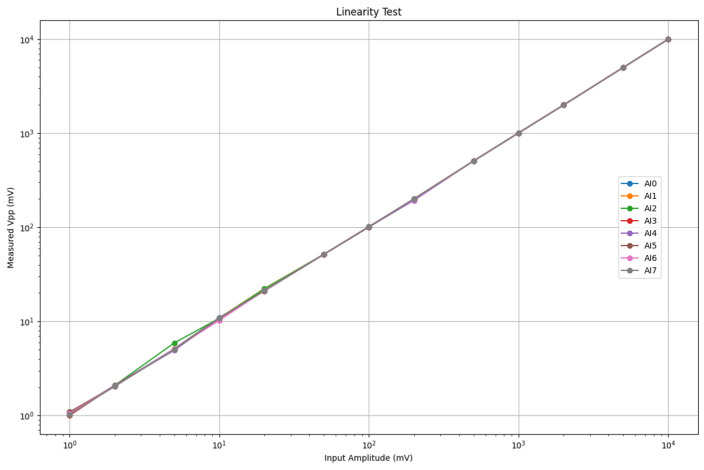
Linearity test results showing measured Vpp versus input Vpp for representative channels across the tested amplitude range.

Results and analysis: The system demonstrated generally good linearity across the majority of the tested amplitude range, particularly for input signals exceeding 100 mVpp (Figure 14). In this higher amplitude region, the measured output Vpp closely tracked the input Vpp, indicating accurate amplitude scaling by the DAQ card. However, noticeable non-linear behavior and inter-channel variations became apparent at lower input amplitudes (specifically in the 1 mV to ~50–100 mV range). Some channels exhibited deviations where the measured Vpp did not perfectly scale with the input Vpp. While minor non-linearities at the lower end of the input range are not uncommon in data acquisition systems, these results suggest that for applications requiring the highest accuracy in measuring very-low-amplitude AE signals (e.g., early damage detection and micro-AE events), potential non-linear effects should be considered. These effects could stem from the DAQ card’s internal amplifiers or ADC characteristics near the bottom of their operational range. For critical low-amplitude measurements, specific channel selection (choosing the most linear channels based on characterization) or software-based amplitude calibration might be necessary to achieve optimal accuracy. The observed inter-channel variability also reinforces the potential benefit of channel-specific calibration if ultimate precision is required across all channels.

#### 3.1.5. Dynamic Range and Signal-to-Noise Ratio

The dynamic range (DR) and signal-to-noise ratio (SNR) are critical metrics quantifying the system’s ability to discern weak signals from background noise and to handle a wide span of signal amplitudes without distortion.

Method:

- Dynamic range (DR): The DR of the DAQ card (without preamplifier) was assessed based on the ratio of the maximum measurable signal (approximated by the RMS value of a near full-scale 10 Vpp, 1 kHz sine wave within the ±10 V range) to the minimum detectable signal (approximated by the RMS noise floor measured with inputs shorted at a 1 MS/s sampling rate, using noise data from Section 3.1.2).
  (2) $$Dynamic\;Range=20\times \log_{10}\left(\frac{v_{max}}{V_{min}}\right)$$
- Signal-to-noise ratio (SNR): The SNR was calculated for a specific high-level input signal. A 10 Vpp, 1 kHz sine wave served as the signal (V_signal_RMS_). The noise (V_noise_RMS_) was taken as the baseline RMS noise measured with inputs shorted (from Section 3.1.2) at two different sampling rates, 1 kS/s and 1 MS/s, allowing the assessment of the impact of the sampling bandwidth on the SNR.
  (3) $$SNR=20\times \log_{10}\left(\frac{v_{signal\text{\_}RMS}}{V_{noise}\text{\_}RMS}\right)$$

**Figure 15:**
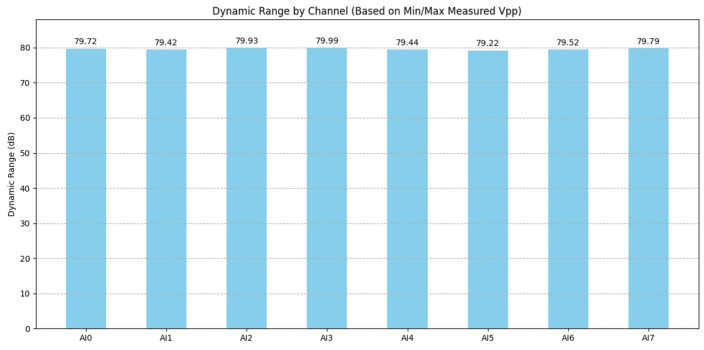
Calculated dynamic range (DR) for each channel of the NI USB-6356 DAQ card.

**Figure 16:**
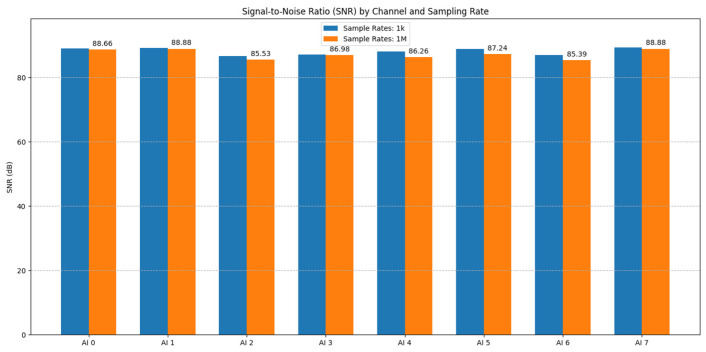
Signal-to-noise ratio (SNR) calculated for a 10 Vpp input signal at 1 kS/s and 1 MS/s sampling rates.

Results and Analysis:

- The calculated dynamic range for the NI USB-6356 DAQ card was found to be approximately 80 dB across the channels (Figure 15). This indicates a strong capability to handle a wide range of signal amplitudes, suitable for capturing both relatively strong AE events and weaker preceding signals, limited primarily by the card’s inherent noise floor and 16-bit resolution. Minor inter-channel variations (e.g., AI0 ~80.1 dB and AI5 ~79.8 dB) were observed. It is important to note that this DR value is based on the measured noise floor and the maximum signal within the ±10 V range; performance with signals in the µV range, potentially limited by linearity (Section 3.1.4) or requiring pre-amplification, warrants further investigation, ideally using lower noise signal sources.
- The signal-to-noise ratio (SNR), evaluated using a high-level 10 Vpp signal, was excellent, exceeding 80 dB for most channels at both 1 kS/s and 1 MS/s sampling rates (Figure 16). This demonstrates the system’s ability to maintain high signal fidelity for strong signals. As expected, the SNR was marginally higher at the lower sampling rate (1 kS/s) compared to the higher rate (1 MS/s), reflecting the integration of a wider noise bandwidth at higher Fs. Inter-channel variations in SNR were also present, mirroring the baseline noise differences. These results confirm the DAQ card’s high intrinsic SNR but also reinforce the practical consideration that system SNR for low-level AE signals will be dominated by the preamplifier noise (Figure 12) and the chosen gain, requiring careful optimization for specific measurement needs.

#### 3.1.6. System-Level Synchronization Performance

Achieving precise time synchronization across multiple channels is paramount for many AE analyses, particularly source localization algorithms that rely on accurately measured arrival time differences (ΔT). While the NI USB-6356 DAQ card incorporates sophisticated hardware synchronization mechanisms (NI-STC3, 100 MHz timebase), promising nanosecond-level precision internally [17], verifying this ultimate precision at the system level (including sensor, AFE, DAQ, USB interface, and Windows host interactions) typically requires specialized external timing instrumentation, which was beyond the scope of this validation study. Therefore, we assessed the overall operational synchronization performance using indirect metrics derived from the acquired data itself, providing a practical evaluation of the timing consistency achievable under normal operating conditions.

Method: Using the data acquired during the channel consistency tests (Section 3.1.3, sine waves 1 kHz–500 kHz), we analyzed inter-channel timing relationships based on the following:

- Cross-correlation: The maximum value of the cross-correlation function computed between all pairs of channels was determined. Values approaching 1 indicate high waveform similarity and phase coherence.
- Relative phase difference: The phase difference (Δφ) between signals acquired on different channels was calculated for each test frequency (as discussed in Section 3.1.3). This Δφ provides a measure of the relative timing error (Δt ≈ Δφ/(2πf)).

**Figure 17:**
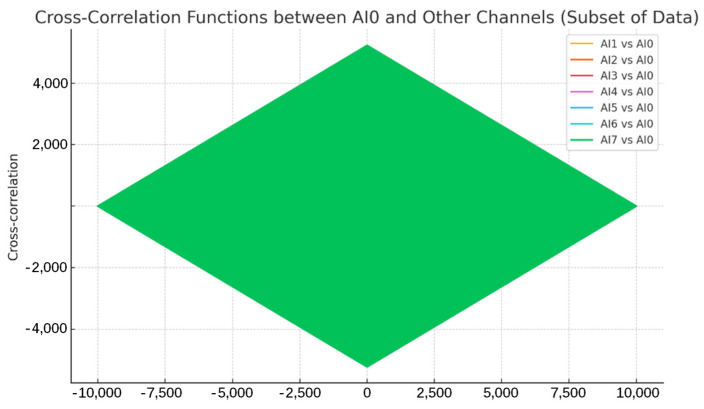
Maximum cross-correlation coefficients calculated between channel pairs for representative test frequencies. Only one color is displayed because of the stacked display.

Results and analysis: The cross-correlation analysis revealed that most channel pairs exhibited maximum correlation coefficients very close to unity (Figure 17), indicating a high degree of similarity in the acquired waveforms and suggesting good overall phase coherence across the system under ideal signal conditions. This provides strong evidence for consistent signal acquisition across the channels. However, a few specific channel pairs showed slightly lower correlation values, warranting caution and suggesting potential minor variations in noise or response for those specific pairs under certain conditions.

**Figure 18:**
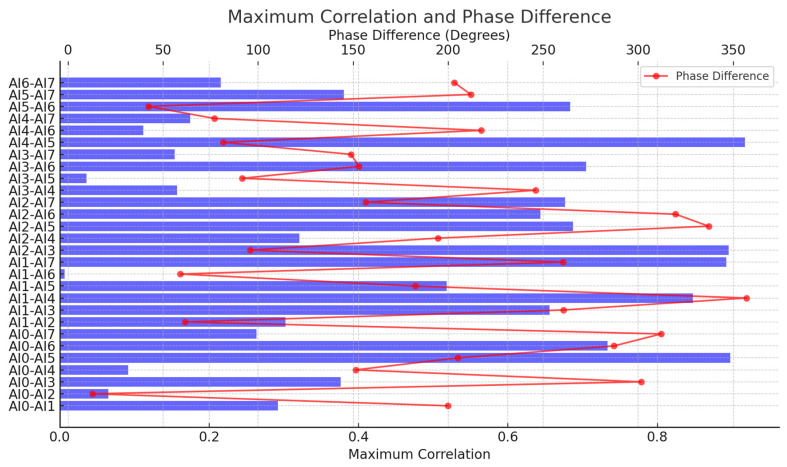
Relative phase differences (degrees or radians) between representative channel pairs across the tested frequency range. The blue bars represent the ‘Maximum Correlation’ values for each channel pair (corresponding to the bottom X-axis).

More revealingly, the relative phase difference analysis (Figure 18, also related to Figure 8 from Section 3.1.3) confirmed the findings from the consistency tests: while minimal at low frequencies, measurable phase differences between channels emerged and increased at higher frequencies. As noted previously, these phase differences correspond to relative timing discrepancies potentially reaching tens of nanoseconds at the highest frequencies tested.

These results collectively indicate that while the system achieves excellent waveform correlation and timing consistency at lower frequencies, the inherent, frequency-dependent phase variations between channels translate to measurable relative timing errors that become non-negligible for applications requiring sub-microsecond ΔT accuracy (common in precise AE source localization). This underscores the practical reality that even with high-quality COTS DAQ hardware featuring internal synchronization, achieving the ultimate timing precision often necessitates software-based post-processing corrections. The characterization performed here provides the necessary data (relative phase/delay information) to implement such corrections effectively within our LabVIEW framework, thereby leveraging the flexibility of the DSP-centric approach to compensate for subtle hardware non-idealities and achieve the high effective synchronization required for advanced AE analysis.

### 3.2. Validation with Acoustic Emission Signals

To demonstrate the system’s capability in practical AE measurement scenarios, validation tests were conducted using pencil lead break (PLB) events. These events generate broadband, transient elastic waves, effectively simulating authentic AE sources.

#### 3.2.1. Experimental Setup

A Hsu–Nielsen source was simulated by breaking a 0.3 mm 2B pencil lead (3 mm protrusion, ~45° angle) on a laboratory desk surface. Four RS-2A AE sensors were coupled to the surface around the PLB site using standard couplant.

#### 3.2.2. Data Acquisition and Software-Based Filtering

**Figure 19:**
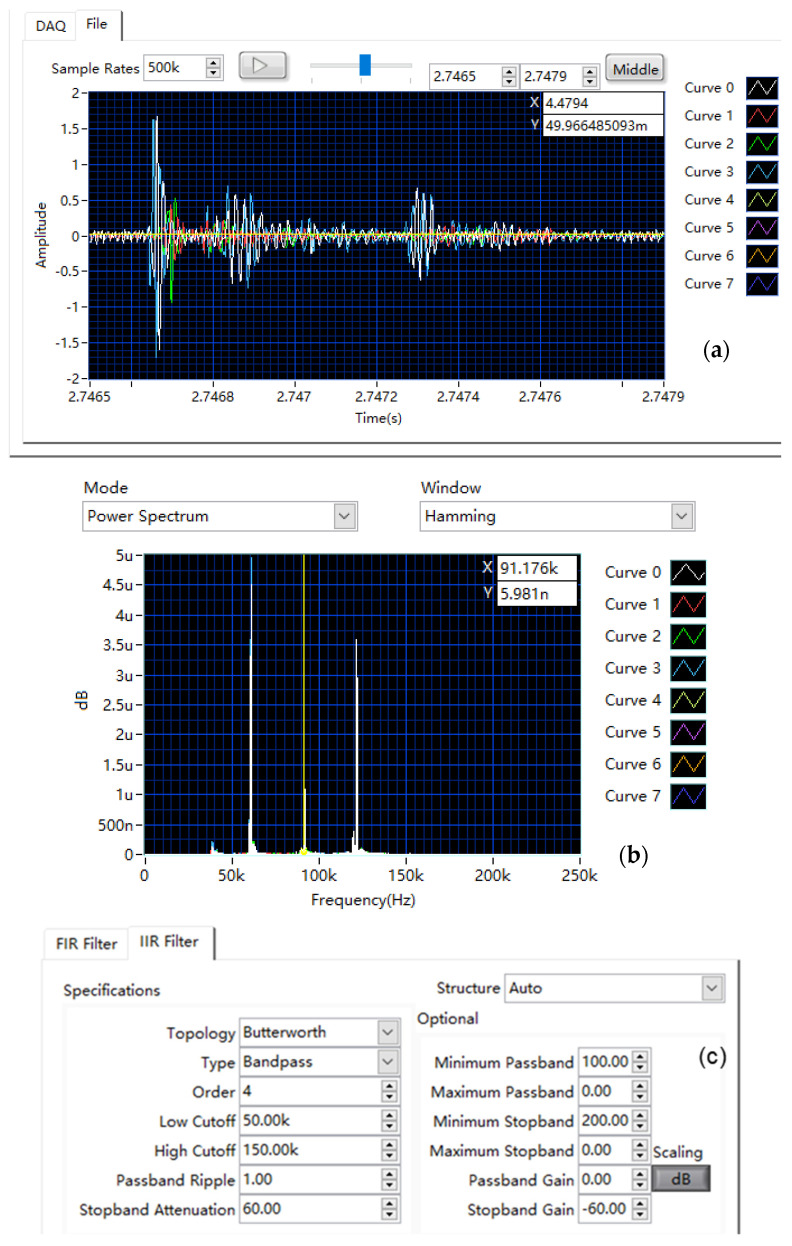
Representative acoustic emission (AE) signal captured from a pencil lead break (PLB) event: (**a**) time-domain waveform after application of the 50 k–150 kHz digital band-pass filter; (**b**) corresponding frequency spectrum (FFT) showing energy concentration within the passband; (**c**) screenshot of the LabVIEW interface section for configuring the digital filter parameters.

Four channels were acquired simultaneously using LabVIEW (Fs = 500 kS/s, ±10 V range, 60 dB gain, 4 s duration). A software-defined 4th-order Butterworth band-pass filter (50–150 kHz), configured via the GUI (Figure 19c), was applied to isolate expected AE frequencies and reject noise.

#### 3.2.3. Representative AE Signal Capture and Analysis

Figure 19 shows representative multi-channel AE signals from a PLB event, including spectral analysis and filter settings.

Time-Domain Analysis: The waveforms (Figure 19a, curves 0–3) exhibit a characteristic sharp onset (t ≈ 2.7466 s) followed by the initial pulse and subsequent decaying wave packets resulting from wave propagation effects (e.g., reflections and mode conversions) within the structure. Variations in arrival times and amplitudes across channels reflect differing propagation paths, crucial for source localization.

Frequency-Domain Analysis: Spectral analysis (FFT with Hamming window, Figure 19b) indicates signal energy concentrated at discrete frequencies. The spectrum features dominant peaks at ~61 kHz (fundamental, f_0_), ~121 kHz (≈2f_0_, likely second harmonic), and a distinct peak at ~91 kHz (interpreted as an independent resonant mode). This multi-peak signature likely arises from combined fundamental, harmonic, and resonant responses excited by the PLB. All major peaks (~61, ~91, ~121 kHz) fall within the filter’s 50–150 kHz passband (Figure 19c), confirming the effective preservation of key signal features.

This multi-peak signature likely arises from combined fundamental, harmonic, and resonant responses excited by the PLB. The observed dominant frequencies (~61 kHz, ~91 kHz, and ~121 kHz) fall within typical ranges reported in the literature for AE signals generated by PLB sources on laboratory setups or specific materials, although the precise spectral signature is known to be highly sensitive to factors such as the specific test object, sensor coupling, and sensor frequency response [4,15]. Importantly, the software-defined filtering approach central to our system (Figure 19c) demonstrates a key advantage here: unlike systems with fixed hardware filters, it allows us to first identify these relevant, empirically observed frequency components and then apply a tailored band-pass filter (50–150 kHz) to effectively isolate them. This adaptability enhances the potential for robust signal feature extraction from diverse AE events. All major peaks (~61, ~91, ~121 kHz) fall within the filter’s 50–150 kHz passband (Figure 19c), confirming the effective preservation of key signal features.

In summary, these results demonstrate the system’s capability for synchronized multi-channel AE acquisition and analysis. The effective use of flexible, software-defined filtering, tailored to the observed signal (here, a 50–150 kHz Butterworth filter detailed in Figure 19c), highlights the system’s adaptability and validates its utility for practical AE measurements.

## 4. Conclusions

This study introduced and rigorously validated a novel methodological approach for multi-channel acoustic emission (AE) data acquisition, designed to enhance flexibility and accessibility while maintaining high performance. By strategically combining digital signal processing (DSP) for filtering with a simplified analog front-end (AFE) and leveraging a high-fidelity commercial synchronous DAQ card (NI USB-6356), our approach effectively circumvents the significant development complexities and costs associated with traditional hardware filtering circuits and bespoke FPGA-based acquisition systems.

Comprehensive performance characterization confirmed the system’s capabilities. We demonstrated accurate frequency capture (<−0.01% relative error) with excellent inter-channel consistency up to 500 kHz. While the core DAQ exhibits low baseline noise and high intrinsic SNR (>80 dB), the preamplifier stage, although essential for amplifying weak signals, remains the dominant system noise source, necessitating careful gain management. The system achieves a dynamic range of approximately 80 dB. Crucially, while excellent waveform correlation was observed, frequency-dependent inter-channel phase variations highlighted the practical need for timing correction in high-precision applications—a task readily amenable to software-based compensation within our flexible architecture. Validation tests using realistic AE signals from pencil lead breaks successfully demonstrated the system’s end-to-end effectiveness in capturing transient events and applying user-defined digital filters.

In conclusion, this work presents more than just a functional AE system; it validates a powerful, DSP-centric paradigm for instrumentation design in this field. We have demonstrated that high-performance, multi-channel AE acquisition is achievable without resorting to complex custom hardware, offering a validated, adaptable, and more readily implementable technological resource. This approach lowers barriers to sophisticated AE monitoring, potentially accelerating research and development across diverse scientific and engineering domains reliant on understanding dynamic material processes, from structural health monitoring and non-destructive testing to materials science and beyond.

The specific features of the proposed system—namely the simplified AFE and the flexible DSP filtering—directly address key requirements found in practical AE monitoring scenarios. For instance, in large-scale SHM applications [9], where deploying numerous sensors can be cost-prohibitive, the simplified AFE design presented here offers a pathway to potentially more economical system architectures. Simultaneously, the flexibility inherent in the software-defined DSP filtering allows the system to be readily adapted to monitor different types of structures or materials, or to optimize performance in varying ambient noise conditions often encountered in machinery diagnostics [8], without necessitating hardware modifications.

### Limitations and Future Work

While this study successfully demonstrated the feasibility and core capabilities of the proposed system, certain limitations warrant discussion and guide future research directions. Firstly, the experimental validation presented herein was primarily conducted using standardized pencil lead break (PLB) tests under controlled laboratory conditions. These tests may not fully represent the signal complexity and challenging noise environments encountered in real-world industrial or field applications. Secondly, as detailed in Section 2.1.3, the employed NI USB-6356 DAQ card lacks onboard hardware anti-aliasing filters. Although mitigation strategies based on sensor/preamp bandwidth and appropriate sampling rate selection relative to the signal bandwidth were effective under the current test conditions, the potential risk of aliasing in environments with strong high-frequency interference cannot be entirely dismissed and requires careful consideration in practical deployments. Thirdly, the simplified AFE, while reducing complexity, might present limitations in terms of the ultimate sensitivity or noise floor compared to specialized, ultra-low-noise commercial AE preamplifiers, particularly for detecting extremely low-amplitude emission events (refer to noise analysis in Section 3.1.2).

Future work will focus on addressing these limitations and further exploring the system’s potential. Priority will be given to validating the system’s performance and robustness in uncontrolled, real-world scenarios, for instance, by applying it to practical engineering problems such as monitoring fatigue crack propagation in metallic bridge components or diagnosing incipient bearing faults in operating industrial machinery. Investigating the scalability of the architecture to a significantly higher number of channels and optimizing data handling for large-scale deployments is another important avenue. Furthermore, the flexible software framework offers opportunities for implementing and evaluating more advanced real-time DSP algorithms, including adaptive filtering techniques, automated feature extraction, machine learning-based event classification, or even basic source localization algorithms. Finally, conducting quantitative benchmark comparisons against established traditional and commercial AE systems would provide valuable insights into the relative performance trade-offs and cost-effectiveness of this DSP-centric approach.

## Figures and Tables

**Table 1:** Parameters for sampling rate and frequency accuracy test.

No.	Test Channel(s)	Test Signal Type	Test Signal Frequency	Test Signal Amplitude	Sampling Rate
1	AI 0~AI 7	Sine Wave	1 kHz	1 Vpp	10 kS/s
2	AI 0~AI 7	Sine Wave	5 kHz	1 Vpp	50 kS/s
3	AI 0~AI 7	Sine Wave	10 kHz	1 Vpp	100 kS/s
4	AI 0~AI 7	Sine Wave	20 kHz	1 Vpp	200 kS/s
5	AI 0~AI 7	Sine Wave	50 kHz	1 Vpp	500 kS/s
6	AI 0~AI 7	Sine Wave	100 kHz	1 Vpp	1 MS/s
7	AI 0~AI 7	Sine Wave	200 kHz	1 Vpp	1 MS/s
8	AI 0~AI 7	Sine Wave	500 kHz	1 Vpp	1.25 MS/s

**Table 2:** Sampling rates used for baseline noise tests (inputs grounded).

No.	Test Channel(s)	Input Condition	Sampling Rate
1	AI 0~AI 7	Directly Grounded	10 kS/s
2	AI 0~AI 7	Directly Grounded	20 kS/s
3	AI 0~AI 7	Directly Grounded	50 kS/s
4	AI 0~AI 7	Directly Grounded	100 kS/s
5	AI 0~AI 7	Directly Grounded	200 kS/s
6	AI 0~AI 7	Directly Grounded	500 kS/s
7	AI 0~AI 7	Directly Grounded	1 MS/s
8	AI 0~AI 7	Directly Grounded	1.25 MS/s

**Table 3:** Test signals used for evaluating inter-channel consistency.

No.	Test Channel(s)	Test Signal Type	Test Signal Frequency	Test Signal Amplitude	Sampling Rate
1	AI 0~AI 7	Sine Wave	1 kHz	1 Vpp	10 kS/s
2	AI 0~AI 7	Sine Wave	5 kHz	1 Vpp	50 kS/s
3	AI 0~AI 7	Sine Wave	10 kHz	1 Vpp	100 kS/s
4	AI 0~AI 7	Sine Wave	20 kHz	1 Vpp	200 kS/s
5	AI 0~AI 7	Sine Wave	50 kHz	1 Vpp	500 kS/s
6	AI 0~AI 7	Sine Wave	100 kHz	1 Vpp	1 MS/s
7	AI 0~AI 7	Sine Wave	200 kHz	1 Vpp	1 MS/s
8	AI 0~AI 7	Sine Wave	500 kHz	1 Vpp	1.25 MS/s

**Table 4:** Input signal amplitudes used for linearity assessment (1 kHz sine wave).

No.	Test Channel(s)	Test Signal Type	Test Signal Frequency	Test Signal Amplitude	Sampling Rate
1	AI 0~AI 7	Sine Wave	1 kHz	1 mVpp, 2 mVpp, 5 mVpp	10 kS/s
2	AI 0~AI 7	Sine Wave	1 kHz	10 mVpp, 20 mVpp, 50 mVpp	10 kS/s
3	AI 0~AI 7	Sine Wave	1 kHz	100 mVpp, 200 mVpp, 500 mVpp	10 kS/s
4	AI 0~AI 7	Sine Wave	1 kHz	1 Vpp, 2 Vpp, 5 Vpp	10 kS/s
5	AI 0~AI 7	Sine Wave	1 kHz	10 Vpp	10 kS/s

## Data Availability

Data are contained within the article.
